# Glycosylation and Crowded Membrane Effects on Influenza Neuraminidase Stability and Dynamics

**DOI:** 10.1021/acs.jpclett.3c02524

**Published:** 2023-10-30

**Authors:** Christian Seitz, İlker Deveci, J. Andrew McCammon

**Affiliations:** † Department of Chemistry and Biochemistry, University of California, San Diego, La Jolla, California 92093, United States; ‡ Department of Pharmacology, University of California, San Diego, La Jolla, California 92093, United States

## Abstract

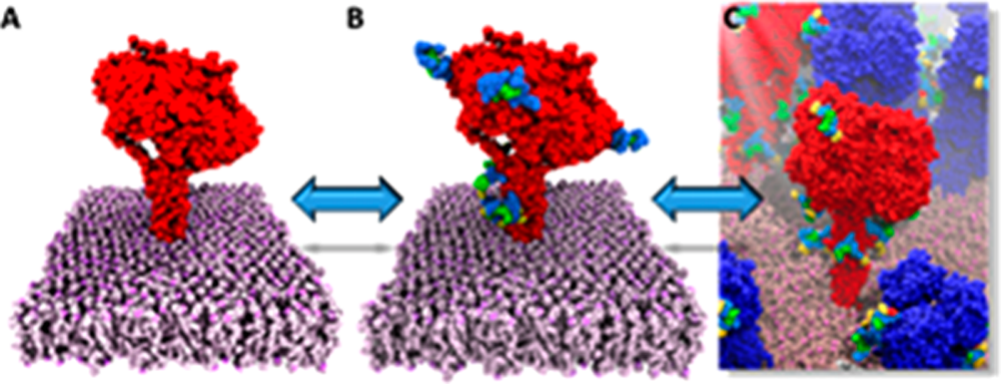

All protein simulations are conducted with varying degrees of simplification, oftentimes with unknown ramifications about how these simplifications affect the interpretability of the results. In this work, we investigated how protein glycosylation and lateral crowding effects modulate an array of properties characterizing the stability and dynamics of influenza neuraminidase. We constructed three systems: (1) glycosylated neuraminidase in a whole virion (i.e., crowded membrane) environment, (2) glycosylated neuraminidase in its own lipid bilayer, and (3) unglycosylated neuraminidase in its own lipid bilayer. We saw that glycans tend to stabilize the protein structure and reduce its conformational flexibility while restricting the solvent movement. Conversely, a crowded membrane environment encouraged exploration of the free energy landscape and a large-scale conformational change, while making the protein structure more compact. Understanding these effects informs what factors one must consider in attempting to recapture the desired level of physical accuracy.

Protein molecular dynamics (MD) simulations has been an exciting field ever since the seminal start in 1977.^1^ However, many of these simulations have been run on single proteins in solvent without any other complicating factors in play to simplify system setup and analysis.^2^ Out of these factors, we selected glycosylation and the protein environment for further study, with the larger goal of indicating when one can simplify these aspects to see a given observable and when one cannot. Glycans are present on about half of all proteins^3^ and play a diverse role biologically, being implicated in molecular recognition,^4^ antibody/drug shielding,^5−7^ protein folding,^8,9^ intracellular transport,^10^ protein clearance,^11^ and more. Glycosylation has been shown to increase protein stability using a variety of experimental and computational techniques,^9,12−20^ but these findings have not been replicated in all protein systems.^21,22^ Decoding the effect glycans have on protein dynamics is similarly ambiguous. Some work has shown that glycosylation may decrease the root-mean-square fluctuations (RMSFs),^23−25^ increase protein dynamics and flexibility,^22^ or reduce global protein mobility^15,26^ or that the dynamics of some regions can be dampened by glycosylation while the dynamics of other regions are promoted by glycosylation,^27^ which can be independent of whether glycans are even found in those regions.^24,28,29^

Environment effects on proteins are also poorly resolved. Proteins are capable of crowding together very effectively,^30^ and this crowding may stabilize protein structures;^31−37^ however, this stabilization effect is dependent on the protein sequence and its shape,^38^ which is complicated by the fact that the protein shape itself can be modified by crowding.^39−41^ However, there is substantial conflicting literature suggesting that protein crowding destabilizes proteins^42−44^ or does not affect their stability at all,^45^ suggesting that whether the environment stabilizes or destabilizes a protein is due to its interactions with the environment^42,43,46−52^ from differences in enthalpic and entropic interactions.^48,53^ Even examining a single protein of interest, some crowding agents can stabilize it^54^ while others destabilize it.^55^ Even less studied is how protein crowding affects the solvent environment. One study showed how water diffusion is faster in systems with more components,^56^ which was contradicted by a different study showing how crowding can reduce the solvent dielectric constant and water self-diffusion^57^ and that systems with a greater degree of crowding slow water diffusion more than less crowded systems.^58^ There is ample room left to investigate how these crowding effects influence protein stability and dynamics.^2^ We shed light on this aspect in this work, investigating how glycosylation and a crowded membrane affect protein stability, protein dynamics, and solvent behavior.

We examined three different systems and will refer to them by their abbreviations throughout the text: a single unglycosylated neuraminidase (NA) tetramer alone in a lipid bilayer, with three replicates of this system simulated (2009-H1N1-ungly); a single glycosylated NA tetramer alone in a lipid bilayer, with three replicates of this system simulated (2009-H1N1-gly); and a single glycosylated NA tetramer in a virus shell with numerous neighboring membrane proteins, with one replicate simulated (2009-H1N1-vir). A representative NA structure is shown in Figure 1, while glycosylation sites are displayed in Table SI1. The 2009-H1N1-gly and 2009-H1N1-ungly systems were constructed and simulated for this work, while the 2009-H1N1-vir system was constructed and simulated previously^59^ and is being further analyzed here. For clarity, the full 2009-H1N1-vir system that was simulated previously^59^ contained 30 NA tetramers spread throughout the viral membrane. We have selected one of these tetramers for analysis in this work; we have termed this tetramer the “2009-H1N1-vir” system. We then extracted that one NA tetramer from the viral membrane at the start of the full virus simulation completed previously and simulated triplicate replicates of it without neighboring proteins in a membrane (the 2009-H1N1-gly system) and then deglycosylated those replicates and simulated them as well (the 2009-H1N1-ungly system). Thus, all simulations started from the exact same protein conformation and were simulated for the exact same length of time using the same MD engine, NAMD, to reduce confounding variables as much as possible. Throughout this work, we will refer to “lateral crowding” or a “crowded membrane” interchangeably; here, we define this to mean the 2009-H1N1-vir environment, focusing on one membrane protein of interest within a model membrane filled with other membrane proteins.

**Figure 1:**
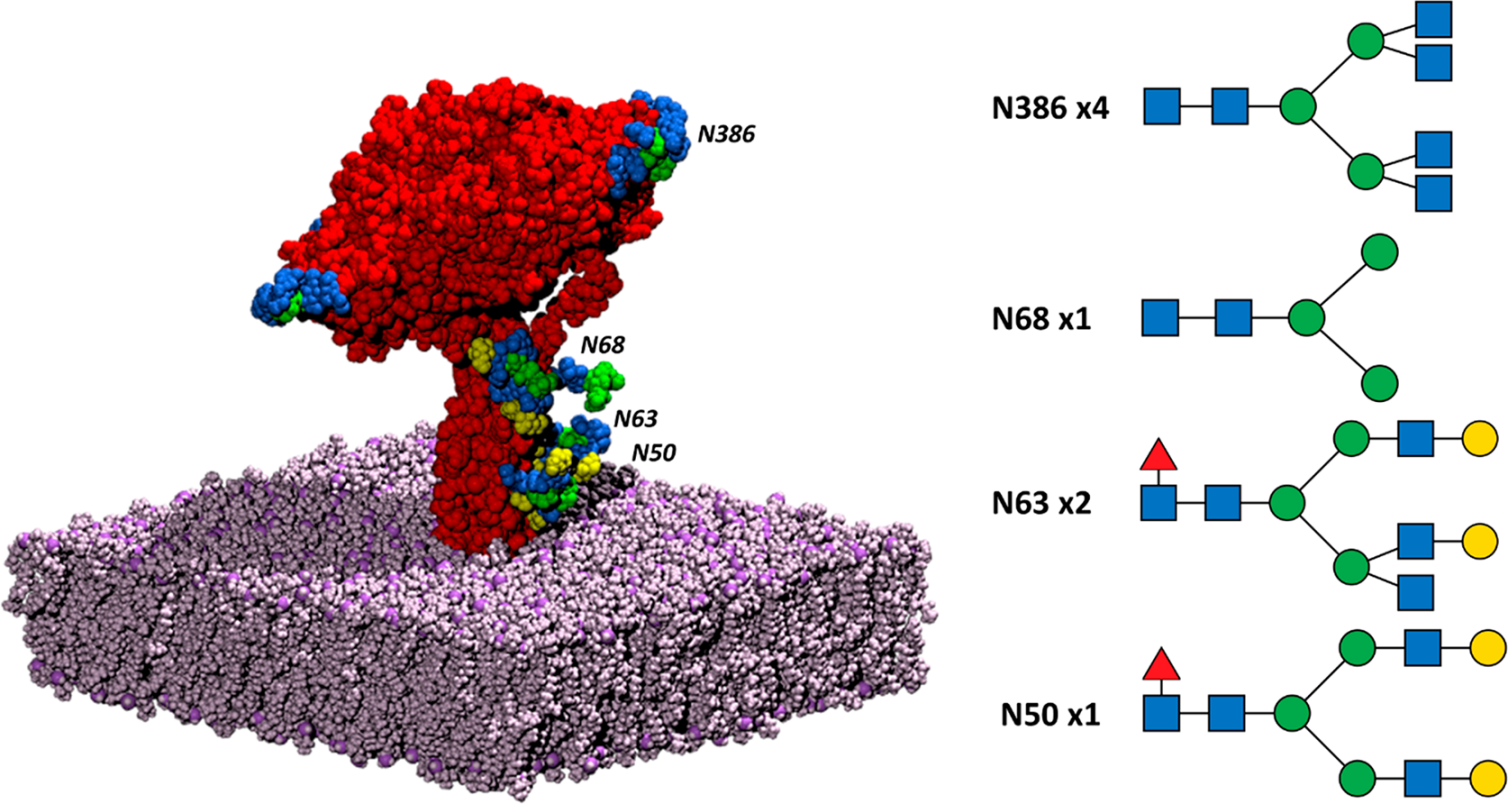
NA system. The NA tetramer is colored red, while the bilayer is in mauve. The glycosylation sites and their glycan structures are also shown; the blue squares represent *N*-acetylglucosamine, the green circles represent mannose, the red triangles represent fucose, and the yellow circles represent galactose. Remembering that there are four monomers in the NA tetramer, there is a glycan at the N386 position in each monomer, with two monomers containing a glycan at N68 and one monomer containing a glycan at N68 and N50. The glycan structures were rendered with GlycoGlyph.^60^

Glycans are chains of atoms that attach to the sides of proteins; intuitively, this should modify the structural stability of the protein, either positively or negatively. This is where our investigations will begin. As a proxy for global protein rigidity, we measured nonpolar interatomic interactions and calculated the energy at which these interactions are excluded; more rigid proteins will retain nonpolar interactions at higher energy cutoffs (see methods in the Supporting Information (SI)^24,61^). We also measured protein compactness through the radius of gyration and stability through root-mean-square deviations (RMSDs). Previous work has shown that glycosylation increases protein rigidity in static structures.^24^ When we examined static structures from our systems, we did not find clear trends in rigidity (Figure SI1A,B). However, examining protein rigidity in dynamic structures showed that the 2009-H1N1-gly and 2009-H1N1-ungly systems have statistically identical rigid cores; thus, glycosylation does not appear to affect rigidity in dynamic proteins. When we compare the environment’s effects on rigidity using RMSD clusters of the simulation frames, we see that the 2009-H1N1-vir system is much more rigid compared to the single protein systems (Figure 2B), while the 2009-H1N1-ungly system is marginally more rigid than the 2009-H1N1-gly system (Figure 2B)—taking into account the standard deviations associated with these measurements, this effect barely reaches statistical significance (Table SI2). Combining these results with Figure 2A, glycosylation may slightly decrease the rigidity of influenza NA, but the effect would be small and may not be seen depending on the methods used. We also see a reduction in the radius of gyration (*R*_g_) of the proteins due to glycosylation, and a further reduction due to a laterally crowded environment (Figure 2C), meaning that they occupy a more compact volume. Previous literature has been divided on this topic: some work shows glycans making proteins more compact,^24,25^ while other work shows that glycans do not affect the compactness of proteins^29^ (and personal correspondence^28^). Glycosylation does not affect the NA stalk stability (Figure 2D) but stabilizes the NA head, as measured by RMSD (Figure 2E). Our data cannot discern whether this stabilization occurs due to glycans stabilizing the NA head at every time point or whether glycans simply increase the time it takes for NA head stabilization to converge. We do not see the same effect for the NA stalk; this may be because the stalk is already less stable so any differences are washed out in the higher RMSD values. In summary, we see that glycosylation slightly decreases the rigidity of NA, makes the NA structure more compact, and stabilizes the NA head (Figure 2). Other work has shown that glycans can stabilize the protein structure^23,25,62^ (Figure SI2), not cause any change in stability,^28,29,63^ or destabilize the structure.^64^ Similar to what we see, some previous work also shows that glycosylation effects on stability may not be consistent throughout the protein of interest and may change depending on which sequon is examined.^65^

**Figure 2:**
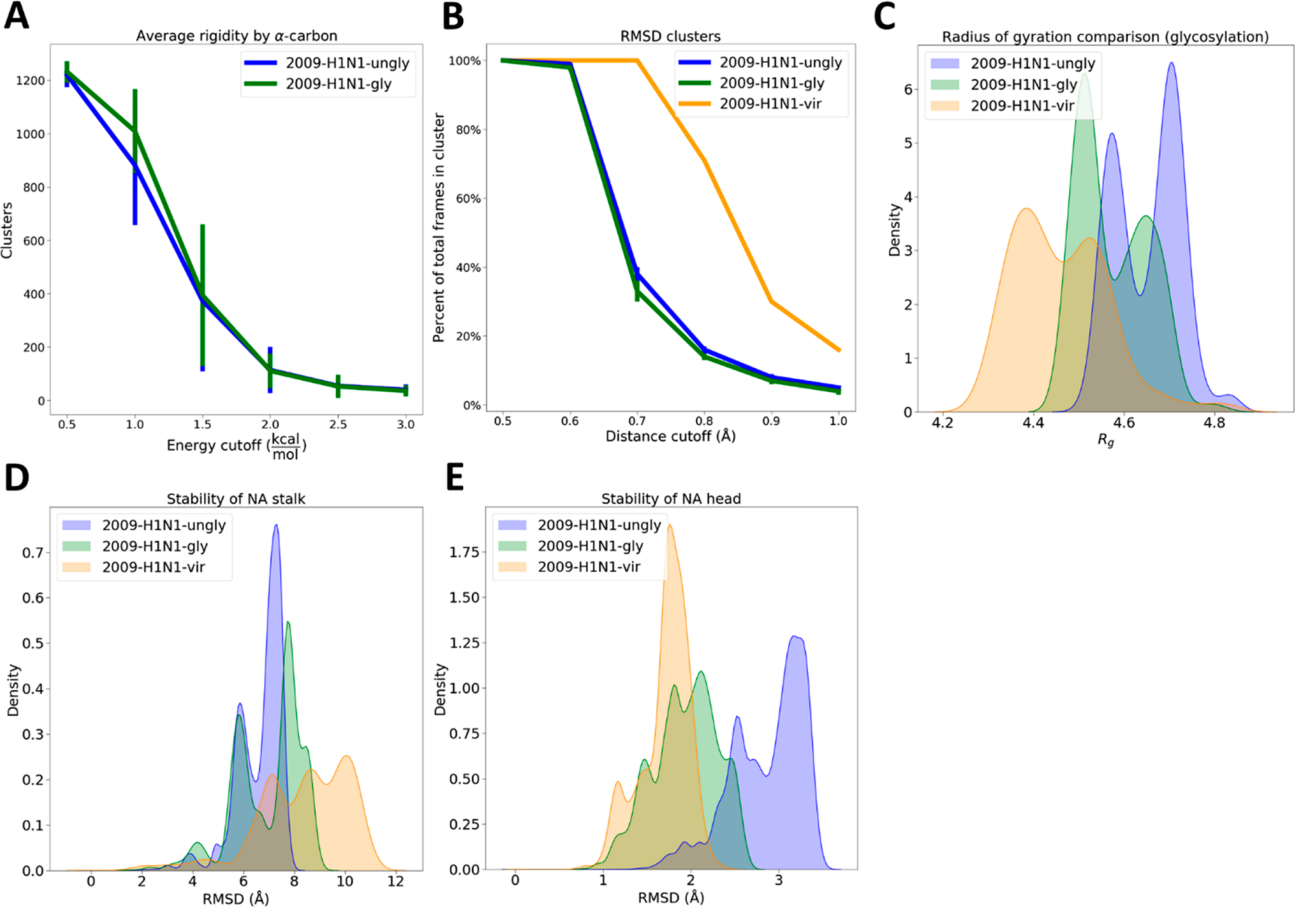
Protein rigidity, compactness, and stability as a function of glycosylation and environment. (A) Glycosylation effects on global protein rigidity, showing the number of α-carbons in the largest rigid cluster that retained nonbonded interactions at different energy cutoffs, along with the sample standard deviation. (B) RMSD clusters of the simulation frames along with the sample standard deviation, showing how many clusters are found as a percentage of total frames at each distance cutoff. (C) Radius of gyration of deglycosylated systems. Here we simulated the 2009-H1N1-gly and 2009-H1N1-vir systems with glycans before removing these glycans for the *R*_g_ calculation. Figure SI1C shows *R*_g_ as a function of time. In Figures 3B and SI5B we show *R*_g_ including the glycans. (D) Stability of the NA stalk as a function of the system. Figure SI1D shows the stalk RMSD as a function of time. (E) Stability of the NA head as a function of the system. Figure SI1E shows the head RMSD as a function of time.

Considering that proteins natively exist in a crowded, chaotic environment, much work has gone into investigating how different crowding conditions and different crowders themselves influence protein stability. There is a much smaller body of literature examining how a crowded membrane, or “lateral crowding”, can affect a membrane protein of interest.^66^ In terms of stability, it does not appear that these membrane protein neighbors influence the stability of NA. They slightly destabilize the NA stalk (Figure 2D) but do not affect the NA head (Figure 2E), nor do they affect protein stiffness (Figure 2B). More differences were seen when investigating protein dynamics in a crowded membrane environment.

After our initial tests on rigidity (Figure 2A,B), we explored protein rigidity further through internal force constants (eigenvalues) and liquid–solid character (Table 1), where higher eigenvalues correlate to stiffer systems and lower Lindemann values correlate to a more solid system. The internal force constants were measured through three methods: (1) principal component analysis (PCA), which reduces the system into a linear combination of coordinates describing the full system dynamics; (2) Gaussian network models (GNMs), which coarse-grain the system into α-carbons connected with an interatomic potential to describe isotropic fluctuations (i.e., independent of the direction of measurement); and (3) anisotropic network models (ANMs), which also coarse-grain the system into α-carbons, this time assuming directional dependence (anisotropy); the Lindemann coefficient was calculated from the PCA space (see methods in the SI(67−75)). Measuring protein stiffness through GNMs and ANMs does not show significant differences due to glycosylation, but an analysis of the principal component space does show that glycans increased the stiffness of NA (Table 1). The crowded membrane environment, and presumably the interprotein connections made therein, then reduce this stiffness to the levels of an unglycosylated system. Considering that stiffer materials will behave more like solids while softer materials will behave more like liquids, we measured the Lindemann coefficient for each of our systems (Table 1). Previous work has shown how phosphorylation can modulate the liquid–solid character of a protein,^76^ but we do not see any significant differences due to glycosylation or the environment (Table 1). This is in line with our previous rigidity work, where we see few, if any, differences in NA rigidity due to glycosylation (Figure 2A,B).

**Table 1:** Stiffness and Solidity of the NA Systems^a^

	PC1 (Å^2^)	GNM	ANM	Lindemann
2009-H1N1-ungly	4080	0.0261	0.00114	0.431 ± 0.060
2009-H1N1-gly	5480	0.0261	0.00114	0.433 ± 0.049
2009-H1N1-vir	4070	N/A	N/A	0.417

a Eigenvalues of the first principal component from PCA and the lowest-frequency mode from the GNM and ANM models are shown, along with the Lindemann coefficient. We did not calculate GNM and ANM modes for the 2009-H1N1-vir system because these would be identical to those of the 2009-H1N1-gly system. The values for the GNM and ANM modes are in arbitrary or relative units, and the Lindemann coefficient does not have a unit.

After seeing that glycans stabilize the NA head (Figure 2E), we wanted to know whether these head glycans would affect the NA dynamics. We studied this through fluctuations through the course of the simulations, the solvent-accessible surface area (SASA), and two studies on entropy: packing entropy, derived from the volume an amino acid occupies divided by its available volume calculated from a static structure, and dihedral entropy, which uses a one-dimensional approximation of the classical coordinate dihedral entropy calculated from the system trajectories (see methods in the SI(77,78)). Due to limitations in sampling, most of these results did not reach the level of statistical significance across our systems. However, considering that we observe the same trends across disparate, unrelated techniques, we will discuss the results here. Similar to previous work,^23−25,79−81^ we see that glycosylation reduces fluctuations of our protein (Figure SI3A). Interestingly, we show a distance dependence of this effect: the dynamics reduction is stronger the closer one gets to the sequon. Other literature, however, has shown that glycosylation can, to a small degree, increase protein dynamics and flexibility^22^ or that the dynamics of some regions can be dampened by glycosylation while the dynamics of other regions is promoted by glycosylation.^27^ We also examined how glycosylation affects entropic properties of the protein; intuitively, one would presume that a reduction in dynamics would be accompanied by a reduction in microstates accessed (and thus a reduction in entropy). This is what we see when examining packing entropy (Figure SI3B) and dihedral entropy (Figure SI3D,E): both are reduced due to glycosylation, and both reductions are stronger at the sequon than in the protein as a whole, in agreement with the RMSF work (Figure SI3A). Previous work has shown that proteins with lower entropy will have lower solvent accessibility.^82−84^ Despite the 2009-H1N1-ungly system having lower solvent accessibility than the 2009-H1N1-gly system (Figure SI3C), there are no global trends in entropy between these two: the 2009-H1N1-ungly system as a whole has slightly higher φ-angle entropy (Figure SI3D) and ψ-angle entropy (Figure SI3E), while they have roughly the same packing entropy (Figure SI3B).

On a broader level, how do these physical changes due to glycosylation affect the biological function of the protein? One study showed how NA can add a glycosylation site to its surface, increasing its enzymatic activity, while removing glycans from NA decreased its sialidase activity, transmission, and virulence.^85^ Thus, adjustment of the glycosylation pattern in NA affects its ability to function. Clearly there are a large number of potential biological reasons for how glycans affect NA fitness *in vivo* which cannot be fully modeled *in silico*. Our work provides a basis to examine the physical differences in NA stability and dynamics due to glycosylation in the hopes that future work may be able to tie phenotypic differences in NA to some of the physical differences outlined here.

If glycosylation reduces the conformational entropy, does the protein environment affect the conformational entropy? We also see that lateral crowding increases the dihedral entropy of the sequon but that this does not appear to affect the dihedral entropy throughout the protein as a whole; in fact, it appears that a crowded protein membrane environment slightly decreases dihedral entropy throughout the protein (Figure SI4). In other words, having a crowded membrane environment increases the conformational entropy of the N-linked sequons while decreasing the conformational entropy of the protein as a whole.

When examining large-scale simulation work, such as work on viral shell simulations,^59^ a persistent question appears: Can we see those same conformational changes and protein characteristics in a simulation with a reduced computational cost? We begin by addressing that significant question here. Some recent work has shown that proteins simulated in a crowded environment can show new motions that are not seen either when the same proteins are simulated individually^59,86^ or when they are simulated in an uncrowded environment,^87^ but transitions between these conformations appear to be slower than in uncrowded environments when they occur.^2^ Other work has shown that protein conformational transitions are not correlated with the protein–protein contacts created in a crowded membrane environment.^59^ We explore how a crowded membrane affects transitions through NA head tilting, a significant conformational change where the NA head swivels on its stalk (see methods in the SI(59)). We find that NA head tilting can be seen in single protein simulations and that this rate of transition is increased by glycans and by using a crowded membrane environment (Figure 3A). The protein–protein contacts inherent in the crowded membrane environment appear to smooth the conformational transitions from a two-peak system to a one-hill system (Figure 3A), although these protein–protein contacts do not appear to be the cause for the increase in the rate of conformational transitions.^59^ It may be due to other environmental differences in our systems, or it may simply be a sampling artifact. Regardless, this rate may be lowered again if a truly crowded environment with free floating proteins were used, as the excluded volume effect will come into play.^88^ In line with this, some other studies have seen suppressed conformational dynamics due to crowding,^52,89,90^ with the caveat that these studies did not examine a membrane crowded with neighboring membrane proteins.

**Figure 3:**
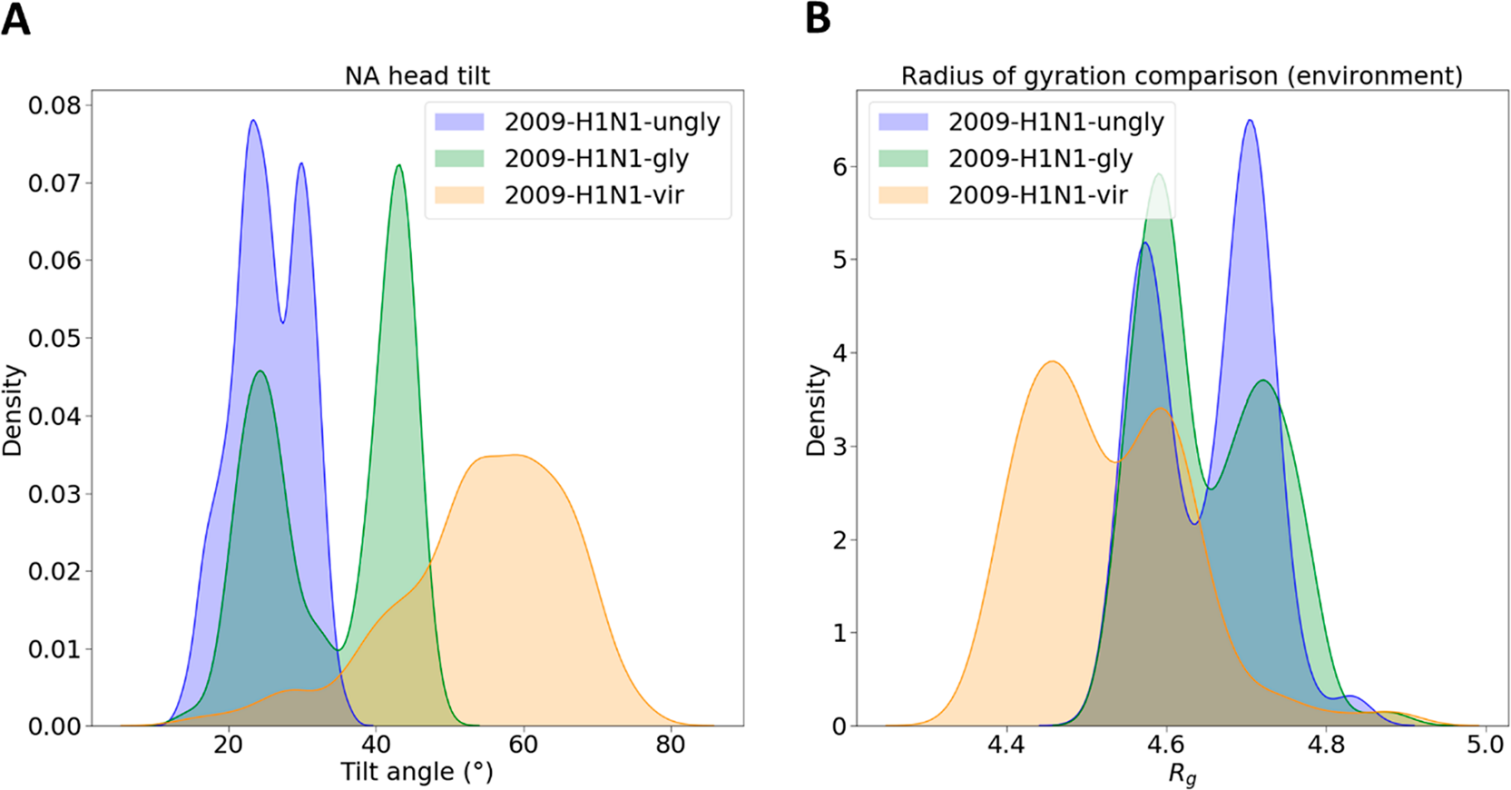
Conformational flexibility and compactness of the NA system. (A) NA head tilt angles. The angle of how much the NA head tilted relative to the stalk was measured. Figure SI5A shows the tilt angle as a function of time. (B) *R*_g_ values for NA. Here we simulated the 2009-H1N1-gly and 2009-H1N1-vir systems with glycans and retained the glycans in the *R*_g_ calculation. Figure SI5B shows *R*_g_ as a function of time. In Figures 2C and SI1C we show *R*_g_ after removal of the glycans.

The 2009-H1N1-vir system is more compact and has a smaller radius of gyration as a whole than the 2009-H1N1-gly system (Figure 3B) due to the presence of protein neighbors; most literature has seen a crowded environment making a protein more compact,^34,39,40,91−93^ while two other studies saw no real difference.^45,94^ A more compact protein would indicate lower entropy; our 2009-H1N1-vir system as a whole contains less dihedral entropy than the 2009-H1N1-gly system (Figure SI4), whereas a system with higher entropy will have higher solvent accessibility.^82−84^ We also saw that our 2009-H1N1-gly system has more dihedral entropy across the protein as a whole than the 2009-H1N1-vir system (Figure SI4) along with a higher solvent accessibility (Figure SI3C). In addition, a more compact protein would indicate faster diffusion because *R*_g_ is inversely proportional to a protein’s instantaneous diffusivity: a higher *R*_g_ means slower diffusion, and vice versa.^95^

Finally, we explored how glycosylation affects sampled space and the harmonic well shape of the first principal component (PC1) from PCA (see methods in the SI(79,96−98)). One previous study found that a protein accesses a smaller amount of conformational space in a crowded environment.^79^ Conversely, we find that lateral crowding increases the amount of the PC1 space NA accessed, with glycans reducing this space accessed (Table 2). This is in line with our RMSF data showing that glycans reduce the global protein range of motion (Figure SI3A). Interestingly, the increase in the PC1 space sampled in the 2009-H1N1-vir system compared to that in the 2009-H1N1-gly system does not correspond to significant differences in the steepness of the harmonic wells corresponding to PC1 (Table 2). Glycans make NA’s PC1 harmonic well more shallow and smooth (Table 2) which may help explain why the 2009-H1N1-gly system displays a larger head tilt than the 2009-H1N1-ungly system. We see glycosylation reducing the PC1 space that NA explores (Table 2); glycosylation reducing conformational space sampled was also seen in a study utilizing 500 ns of MD sampling,^28^ comparable to our 441 ns of sampling, although a different study utilizing 75+ μs of MD sampling saw that glycosylation does not affect the conformational space that one protein of interest can access.^99^ This suggests that glycosylation may slow the exploration of the PC1/free energy but that an exhaustive sampling regime may find similar spaces explored irrespective of glycosylation.

**Table 2:** Internal Dynamics of the NA Systems as Measured through PCA^a^

	PC1 space sampled (%)	*k* (kJ/mol^–1^·nm^–2^)	*a*	τ (ns)	ζ (amu/ns)
2009-H1N1-ungly	100	9.46 × 10^–6^	1.15	47.1	438
2009-H1N1-gly	83	5.90 × 10^–6^	1.28	48.7	268
2009-H1N1-vir	94	6.09 × 10^–6^	1.09	51.5	313

a The 2009-H1N1-ungly system was set as the reference space sampled, and the sampling of the other two systems was measured relative to this. *k* is the harmonic force constant; larger *k* values indicate deeper and sharper energy wells explored by the systems. The internal friction coefficient ζ indicates diffusion through the free energy surface; larger ζ values indicate more friction, i.e., a rougher potential energy surface, which equates to slower diffusion through that surface. The *a* and τ parameters are included for completeness, as they are needed for full computation.

Intuitively, glycosylation will increase the solvent accessibility of a protein by increasing its surface area, which we see in our work (Figure SI3C). Increased solvent accessibility should result in a restriction of solvent motion;^58^ we see a corresponding, albeit small, restriction in solvent motion due to glycosylation (Table SI3). However, this was calculated including all of the solvent around the protein, so most of the “signal” of the decrease in solvent motion due to glycosylation might have been lost in the noise. We carried out more detailed calculations honing in on how glycans affected the entropy and energy of the solvent through grid inhomogeneous solvation theory (see methods in the SI(100−103)). We see that glycosylation reduces the solvent entropy and orders the water to a certain extent while reducing the amount of free water in the vicinity of the glycan (Table 3). Thus glycans can slow down water passage near the protein surface.

**Table 3:** Per-Water Thermodynamic Properties of the Solvent in the Glycan-Adjacent Regions^a^

	*TS*_trans_ (kcal/mol)	*TS*_orient_ (kcal/mol)	*E*_sw_ (kcal/mol)	*E*_ww_ (kcal/mol)
2009-H1N1-ungly	–0.05 ± 0.04	–0.06 ± 0.04	–0.48 ± 0.31	–8.89 ± 2.26
2009-H1N1-gly	–0.35 ± 0.10	–0.36 ± 0.09	–1.89 ± 0.54	–2.11 ± 0.48
difference	–0.30 ± 0.08	–0.31 ± 0.10	–1.41 ± 0.44	6.78 ± 1.83

a Glycans restrict water movement and coordinate waters. *TS*_trans_ and *TS*_orient_ are the translational and rotational entropies, respectively, in the protein frame of reference. More negative values indicate lower entropy, thus showing that water is more constrained positionally and orientationally, respectively. Bulk water entropy values are set to be 0. *E*_sw_ and *E*_ww_ are interaction energies per water molecule for solute–water and water–water interactions, respectively. More negative *E*_sw_ values indicate more favorable interactions between the water and the solute and thus show whether water coordinates to the solute. These values would be more negative near charged side chains and less negative near nonpolar side chains. More negative *E*_ww_ values indicate more favorable interactions between water and the rest of the water in the solvent. These values would be more negative where water is free and acting like bulk water and less negative where water is sequestered. We averaged solvent entropy and energy around each of the glycans and subtracted the entropy and energy from the same space on the 2009-H1N1-ungly construct from the 2009-H1N1-gly construct to arrive at a difference. The sample standard deviation is also presented.

To decode the effects of glycans and lateral protein crowding on influenza NA, we examined three systems with each one differing by one variable: two systems are identical except for the presence/absence of glycans, and two systems (including one of the two above) are identical except for the environment they were studied in. This allows us to do a direct comparison of the effects that glycosylation and membrane crowding have on protein stability and dynamics. We found that glycans generally increase the stability, stiffness, and rigidity of NA while reducing fluctuations, entropy, and the sampling of configuration space. Glycans also increase the degree of large-scale conformational change in NA. A crowded membrane protein environment increases the entropy of the glycan sequons and may promote a large-scale conformational change. At the solvent level, glycans restrict water movement. Understanding how glycosylation and protein environment affect a protein’s characteristics informs the scenarios when it is acceptable to reduce the complexity of a protein model and still recover its native characteristics and when a reduction in complexity is not valid. There is ample space for future work to investigate how translatable these results are to other systems and how well (or poorly) they hold up when creating larger protein environments that contain more realistic biological environments.

## Supporting Information Available

Methods for system creation, running, and analysis; N-linked residues connecting each of the glycans to the protein (Table SI1); protein rigidity, compactness, and stability as a function of glycosylation and environment (Figure SI1); rigidity of the systems as measured by RMSD clusters (Table SI2); stability of SARS and SARS2 systems as a function of glycosylation (Figure SI2); protein fluctuations, entropy, and solvent accessibility as a function of glycosylation (Figure SI3); dihedral entropy of the NA structure as a function of protein environment (Figure SI4); conformational flexibility and compactness of the NA system (Figure SI5); effective viscosity of the solvent in the NA systems (Table SI3) (PDF)
Transparent Peer Review report available (PDF)
