# Supplementary material for: Glycosylation and Crowded Membrane Effects on Influenza Neuraminidase Stability and Dynamics
Source: J Phys Chem Lett. 2023 Oct 30;14(44):9926–34. doi: 10.1021/acs.jpclett.3c02524 (PMC10641874; doi:10.1021/acs.jpclett.3c02524)
Supplement: Supplementary file 2 — jz3c02524_si_002.pdf [file jz3c02524_si_002.pdf]

jz-2023-02524e.R1

Name: Peer Review Information for "Glycosylation and Crowded Membrane Effects on Influenza Neuraminidase Stability and Dynamics"

First Round of Reviewer Comments

Reviewer: 1

Comments to the Author

This study investigates how glycosylation and membrane crowding influence the dynamics of influenza neuraminidase (NA). Three systems were explored: 1) NA with glycan attachments, 2) NA without glycosylation, and 3) NA extracted from a virus shell surrounded by neighboring NA proteins. The authors analyzed key physical properties including RMSF, radius of gyration, PCA, SASA etc. They found that glycans enhance protein stability, reducing flexibility and restricting solvent movement. Additionally, crowded membranes induce significant conformational changes, resulting in a more compact protein structure.

While crowding effects have been extensively studied in various previous research, the impact of neighboring proteins on the membrane is less explored. This study is significant in providing insightful understanding of the physical features influenced by glycosylation and crowded membrane using an influenza virus protein as an example. It represents a novel contribution to the field. I strongly recommend this work for publication in The Journal of Physical Chemistry Letters. The scientific background and research plan are well presented in this manuscript; however, I suggest the authors address the following points to further strengthen the manuscript.

Regarding the 2009-H1N1-vir system, where there are multiple NA proteins on the virus surface, did the authors analyze all of them, or did the authors select specific ones for analysis? The method section in the SI should provide details on how the selection process was carried out. Additionally, it would be beneficial to include more information about the 2009-H1N1-vir system, such as the distance between neighboring NA proteins on the virus shell. Since this study explored crowded membrane effects, the proximity of neighboring membrane proteins may have an impact on the conclusions drawn.

Regarding Figure 2A, the authors noted that the 2009-H1N1-ungly system is slightly more stable at low energy cutoffs (≤ 1.0 kcal/mol) due to the exclusion of flexible glycans. However, as the energy cutoff is increased (≥ 1.0 kcal/mol), the 2009-H1N1-gly system becomes a more stable, rigid structure. Yet, when

the energy cutoff is larger than 2.3 kcal/mol, the 2009-H1N1-ungly system is once again slightly more stable than the 2009-H1N1-gly system. The authors did not provide an explanation for this observation.

In Table 1, the authors mentioned that "the principal component space does show that glycans increased the stiffness of NA." However, there is no explanation provided for the reduction in PC1 observed in the 2009-H1N1-vir system.

In Figure 3, could the authors elucidate why 2009-H1N1-gly and 2009-H1N1-ungly exhibit two peaks, while 2009-H1N1-vir does not?

Reviewer: 2

Comments to the Author

The manuscript by Seitz et al. presents a study that employs MD simulations to examine the impact of glycosylation and a crowded membrane environment on influenza neuraminidase (NA). Their MD simulations uncovered several noteworthy findings. Firstly, they observed that glycans enhance the stability, stiffness, and rigidity of NA, leading to a greater degree of large-scale conformational changes within the protein. Furthermore, in a crowded membrane protein environment, an increase in the entropy of the glycan sequons was observed. However, the study primarily focuses on a single protein, which raises questions about the generalizability of these findings to other proteins. To ascertain the broader applicability, it is advisable to explore these effects across multiple proteins or elucidate the underlying principles driving these dynamics changes. Another critical consideration is the need to establish the physical significance and biological relevance of the observed dynamics changes. It is essential to confirm that these changes are not trivial or spurious and that they remain consistent over longer simulation times or under varying conditions. While this study represents a solid contribution, it may require further substantiation to clearly demonstrate a "significant advance" in the field.

Author's Response to Peer Review Comments:

Reviewer 1:

This study investigates how glycosylation and membrane crowding influence the dynamics of influenza neuraminidase (NA). Three systems were explored: 1) NA with glycan attachments, 2) NA without glycosylation, and 3) NA extracted from a virus shell surrounded by neighboring NA proteins. The authors analyzed key physical properties including RMSF, radius of gyration, PCA, SASA etc. They found that glycans enhance protein stability, reducing flexibility and restricting solvent movement. Additionally,

crowded membranes induce significant conformational changes, resulting in a more compact protein structure.

While crowding effects have been extensively studied in various previous research, the impact of neighboring proteins on the membrane is less explored. This study is significant in providing insightful understanding of the physical features influenced by glycosylation and crowded membrane using an influenza virus protein as an example. It represents a novel contribution to the field. I strongly recommend this work for publication in The Journal of Physical Chemistry Letters. The scientific background and research plan are well presented in this manuscript; however, I suggest the authors address the following points to further strengthen the manuscript.

First of all, we would like to thank this reviewer for taking the time to examine this manuscript. From their questions below, it is clear that they have thought critically about the science presented here, and have asked detailed questions about very specific points that can be improved in this manuscript that would not be apparent if one were skimming the paper. Answering these thoughtful questions has allowed us to strengthen the manuscript, and we would like to thank this reviewer for that.

1) *Regarding the 2009-H1N1-vir system, where there are multiple NA proteins on the virus surface, did the authors analyze all of them, or did the authors select specific ones for analysis? The method section in the SI should provide details on how the selection process was carried out. Additionally, it would be beneficial to include more information about the 2009-H1N1-vir system, such as the distance between neighboring NA proteins on the virus shell. Since this study explored crowded membrane effects, the proximity of neighboring membrane proteins may have an impact on the conclusions drawn.*

   It is important to be clear with which systems are being used in our comparisons, and we agree that this currently is not as clear as it should be. We thank this reviewer for pointing that out, and for the chance to clarify these points for future readers. We have added information to the main text and to the SI clarifying these points. To summarize what we have added, the 2009H1N1-vir system was simulated previously with 30 NA tetramers spread over the membrane surface. We chose one tetramer that exhibited a moderate amount of a conformational change (NA head tilting) and extracted its starting conformation to use in single protein simulations (the

   2009-H1N1-gly system). We then deglycosylated the 2009-H1N1-gly system, thus creating the 2009-H1N1-ungly system, and simulated those copies as well. This means that all systems presented here, namely the 2009-H1N1-vir system, the 2009-H1N1-gly system, and the 2009H1N1-ungly system, all had the exact same starting protein conformation and were simulated for the exact same length of time. This was done to reduce confounding variables as much as possible and make the conclusions easier to understand.

   Although there are several differences between a simulation of a whole virus and a simulation of a single protein, such as the much larger solvent volume present in the former simulation, the

proximity of neighboring membrane proteins in a whole virus simulation is likely the primary cause for the effects presented here. As mentioned above, the NA tetramer, comprising the 2009-H1N1-vir system analyzed in the work presented here, was originally simulated in this work: https://pubs.acs.org/doi/full/10.1021/acscentsci.2c00981. We have added information to the SI directing the reader to Figure 6, Figure S26 and Figure S27 of that work, which details how the NA tetramers in the viral membrane are making several contacts with each other. Thus, they are dynamically breaking, and sometimes forming, contacts with each other – they are not simply coexisting without interacting with each other.

2) *Regarding Figure 2A, the authors noted that the 2009-H1N1-ungly system is slightly more stable at low energy cutoffs (≤ 1.0 kcal/mol) due to the exclusion of flexible glycans. However, as the energy cutoff is increased (≥ 1.0 kcal/mol), the 2009-H1N1-gly system becomes a more stable, rigid structure. Yet, when the energy cutoff is larger than 2.3 kcal/mol, the 2009-H1N1-ungly system is once again slightly more stable than the 2009-H1N1-gly system. The authors did not provide an explanation for this observation.*

This is a great question, one that requires careful thought. The reviewer is bringing up a question regarding our use of the FLEXOME software. This is new software, so its use cases are still being explored. We had already measured the protein rigidity of static structures at 298 K (now Figure SI2A), which showed the 2009-H1N1-ungly system being more rigid than the 2009H1N1-gly system at lower energy cutoffs before becoming less rigid at higher energy cutoffs. Explaining this odd "rigidity switch" is what the reviewer is astutely asking here. To appropriately answer the reviewer's question, we reached out to one of the developers of FLEXOME, Prof. Stephen Wells. Prof. Wells suggested taking our structures and relaxing them at 0 K before performing the rigidity analysis. Slowly relaxing our structures to 0 K should allow the hydrogen bonds present to move toward energy minima, instead of being captured in some dynamic, non-minima bond length as would be expected in a single MD frame. This would in theory would improve the performance of FLEXOME and strengthen our findings. We did this in Figure SI2B; however, this still showed an odd switch in rigidity that we could not explain. Instead of examining single structures, we decided to sample structures throughout the MD simulations, the idea being that sampling enough structures would create an average O – H…O hydrogen bond geometry that would be more robust than using one geometry from one structure. We now show this in Figure 2A: Sampling enough frames shows that the odd "rigidity switch" we saw previously was a factor of not having enough sampling, and that there should be minimal differences in the system rigidity due to glycosylation. This is now consistent with our findings in Figure 2B, using RMSD, that also shows minimal differences in protein rigidity due to glycosylation. Thus, we would like to thank this reviewer for catching this detail; exploring the issue the reviewer brought up allowed us to make a more consistent story.

3) *In Table 1, the authors mentioned that "the principal component space does show that glycans increased the stiffness of NA." However, there is no explanation provided for the reduction in PC1 observed in the 2009-H1N1-vir system.*

We thank the reviewer for catching this, as this point is not explained in the text. As the reviewer notes, Table 1 shows that glycans increase the stiffness of the protein as observed through principal component analysis. Examining the same protein in a crowded membrane environment then reduces the protein stiffness, presumably through the interprotein connections made. We have added this sentence to the text: “The crowded membrane environment, and presumably the interprotein connections made therein, then reduces this stiffness to the levels of an unglycosylated system.”

4) *In Figure 3, could the authors elucidate why 2009-H1N1-gly and 2009-H1N1-ungly exhibit two peaks, while 2009-H1N1-vir does not?*

This is an excellent question. To summarize, the reviewer would like to know why the degree of the conformational change analyzed in Figure 3, NA head tilting, shows two distinct peaks for the 2009-H1N1-gly and 2009-H1N1-ungly systems but not for the 2009-H1N1-vir system. The 2009-H1N1-gly and 2009-H1N1-ungly systems do not contain external “influences”, such as neighboring proteins, and they both follow a conformational distribution with two peaks. The 2009-H1N1-vir system can make contacts with neighboring proteins, which likely affect its path from one conformation to another. Thus, the neighboring proteins smooth out the path of conformational change, wiping out the two peaks seen in the 2009-H1N1-gly and 2009-H1N1ungly systems. We have updated the text to reflect this, and thank the reviewer for the opportunity to clarify this point.

Reviewer 2:

The manuscript by Seitz et al. presents a study that employs MD simulations to examine the impact of glycosylation and a crowded membrane environment on influenza neuraminidase (NA). Their MD simulations uncovered several noteworthy findings. Firstly, they observed that glycans enhance the stability, stiffness, and rigidity of NA, leading to a greater degree of large-scale conformational changes within the protein. Furthermore, in a crowded membrane protein environment, an increase in the entropy of the glycan sequons was observed.

We thank the reviewer for taking the time to examine this paper, and to give us the opportunity to improve it.

1) *However, the study primarily focuses on a single protein, which raises questions about the generalizability of these findings to other proteins. To ascertain the broader applicability, it is advisable to explore these effects across multiple proteins or elucidate the underlying principles driving these dynamics changes…It is essential to confirm that these changes are not trivial or spurious and that they remain consistent over longer simulation times or under varying conditions. While this study represents a solid contribution, it may require further substantiation to clearly demonstrate a "significant advance" in the field.*

Our original goal when designing this project was to craft a series of broad, generalizable rules governing how glycans and a crowded membrane environment affect protein stability and dynamics. As we carried out the project and gathered data, we concluded that our original goal would not be possible to achieve. We will address both points from this reviewer below.

Do these results remain consistent over longer simulation times?

This is consistently a useful question to ask in regards to protein simulations. These simulations appear to have converged, as measured by RMSD (Figure SI1C and Figure SI1D).

Thus, we expect these results to remain consistent over longer simulation times.

Furthermore, the work presented here is an extension of a significant amount of prior work. It took over five years to construct the influenza membrane (Durrant et al, ACS Central Science 2020) and then simulate it for ~450 ns (Casalino et al, ACS Central Science 2022). This system contained ~160 million atoms while using 4,096 physical nodes (28,672 processors) on the TITAN supercomputer at Oak Ridge National Laboratory, at a speed of ~13.83 ns/day (Casalino et al, ACS Central Science 2022). In other words, this simulation took about a quarter of the total nodes on what was one of the 10 fastest supercomputers in the world at the time the simulation took place. This represents an enormous expenditure of computing resources; it is from this system that we extracted one NA protein to be used as the 2009-H1N1-vir system presented here. Thus, it is not readily feasible to extend the simulation of the ~160 million atom influenza membrane, meaning it is also not readily feasible to extend the simulation of the 2009-H1N1-vir system presented here. While we could extend the simulations of the 2009-H1N1-gly and 2009-H1N1-ungly systems as these contain only one protein in a bilayer, we would not be able to make direct comparisons with the 2009-H1N1-vir system anymore as they would contain uneven amounts of sampling.

Do these results remain consistent under varying conditions? Can these results be generalized to other systems?

This is another important question to be asking. There is limited information in the literature on how a crowded membrane environment affects proteins. However, there is a body of literature on how glycans affect proteins. Unfortunately, from looking this body of work one sees that the effects glycans have on proteins varies considerably across the proteins examined. Let's look at some simple metrics. In some systems glycosylation has been shown to increase protein stability (Shental-Bechor et al, PNAS 2008, Joao and Dwek, European Journal of Biochemistry 1993, Bonzom et al, AMB Express 2019 and several others) but in other systems this has not been replicated (Gavrilov et al, Journal of Physical Chemistry Letters 2015 and Danwen et al, Pharmaceutical Research 2016). Some work has shown that glycans can dampen protein fluctuations in some regions but enhance fluctuations in other regions (Acharya et al, Chemical Communications 2021) and that glycans can either promote or inhibit protein fluctuations on regions of the protein that are not glycosylated (Ramakrishnan et al, FEBS Journal 2023, Škulj et al, Computational and Structural Biotechnology Journal 2022, Pol-Fachin et al, Glycoconjugate Journal 2016). If we examine how glycans affect such simple metrics as stability and fluctuations and do not find any

general rules, we do not feel comfortable attempting to generalize the results we present here to other systems that they likely cannot be replicated in. Thus, we have taken great care in this manuscript to situate our results within the literature without falsely guaranteeing that these results can be generalized to other systems. Our aim with this work is twofold. Our first goal is to present a series of wide-ranging analyses and the means to reproduce them in new systems. This will hopefully lead to our second goal, where the very detailed analyses presented here and elsewhere will lead to a more complete and generalizable understanding of how glycans and a crowded membrane environment affect protein dynamics and stability, which does not appear to be feasible with our current technology.

2) *Another critical consideration is the need to establish the physical significance and biological relevance of the observed dynamics changes.*

We would like to thank the reviewer for bringing up this point, and for the opportunity to address it as we did not appropriately address the biological relevance of our findings. To address this, we have added the following paragraph to the manuscript:

"On a broader level, how do these physical changes due to glycosylation affect the biological function of the protein? One study showed how NA can add a glycosylation site to its surface, increasing its enzymatic activity, while removing glycans from NA decreased its sialidase activity, transmission, and virulence. (85) Thus, adjusting the glycosylation pattern in NA affects its ability to function. Clearly there are a large number of potential biological reasons for how glycans affect NA fitness in vivo which cannot be fully modeled in silico. Our work provides a basis examining the physical differences in NA stability and dynamics due to glycosylation in the hopes that future work may be able to tie phenotypic differences in NA to some of the physical differences outlined here."
